# Analysis of prognostic factors for in-hospital mortality in patients with unplanned re-exploration after cardiovascular surgery

**DOI:** 10.1186/s13019-022-01825-7

**Published:** 2022-04-23

**Authors:** Jianying Deng, Qianjin Zhong

**Affiliations:** 1 Department of Cardiovascular Surgery, Chongqing Kanghua Zhonglian Cardiovascular Hospital, 168# Haier Street, Jiangbei District, Chongqing, 400015 China; 2 Department of Cardiovascular Surgery, Army Medical Center of PLA, Chongqing, 400020 China

**Keywords:** Cardiovascular surgery, Re-exploration, In-hospital mortality, Prognostic factor

## Abstract

**Objective:**

To explore the prognostic factors for in-hospital mortality in patients with unplanned re-exploration after cardiovascular surgery.

**Methods:**

We retrospectively analyzed the data of 100 patients who underwent unplanned re-exploration after cardiovascular surgery in our hospital between May 2010 and May 2020. There were 77 males and 23 females, aged (55.1 ± 15.2) years. Demographic characteristics, surgical information, perioperative complications were collected to establish a database. These patients were divided into surviving and non-surviving groups according to in-hospital mortality. Logistic regression was used for multivariable analysis to explore the prognostic factors of in-hospital mortality. These statistically significant indicators were selected for drawing the receiver operating characteristic curve of the evaluation model, calculating the area under the curve (AUC) and evaluating the effectiveness of the new model with Hosmer–Lemeshow C-statistic.

**Results:**

In-hospital mortality in patients with unplanned re-exploration after cardiovascular surgery was 26.0% (26/100). Multivariate logistics regression revealed that the operation time of unplanned re-exploration, the worst blood creatinine value within 48 h before the re-exploration, the worst lactate value within 24 h after the re-exploration, cardiac insufficiency, respiratory insufficiency, and acute kidney injury were independent prognostic factors (*P* < 0.05). The AUC of the new assessment model constituted by these prognostic factors was 0.910, and the Hosmer–Lemeshow C-statistic was 4.153 (*P* = 0.762).

**Conclusions:**

Operation time of unplanned re-exploration, worst serum creatinine value within 48 h before re-exploration, worst lactate value within 24 h after re-exploration, cardiac insufficiency, respiratory insufficiency, and acute kidney injury are the main prognostic factors for in-hospital mortality in patients with unplanned re-exploration after cardiovascular surgery. Identifying these prognostic factors can effectively facilitate preventive measures and improve patient outcomes.

## Introduction

Unplanned re-exploration is a serious adverse event after cardiovascular surgery, which will prolong the patient’s hospital stay, ICU stay, mechanical ventilation time, and ultimately lead to an increase in the incidence of complications [1–3]. Studies have shown that in-hospital mortality in patients undergoing unplanned re-exploration after cardiovascular surgery is 8–25% [4], which is much higher than in patients who did not undergo re-exploration. A large number of previous studies have mostly focused on patients with severe bleeding or cardiac arrest after cardiac surgery, focusing on factors associated with unplanned re-exploration after cardiovascular surgery. These results suggest that advanced age, low body mass index, longer cardiopulmonary bypass time, and secondary cardiac surgery are independent risk factors for unplanned re-exploration thoracotomy after cardiovascular surgery [5–7]. On the basis of previous research, combined with the characteristics of our hospital, we took patients who underwent unplanned re-exploration thoracotomy after cardiacvascular surgery as the research objectives, and explored the prognostic factors of in-hospital mortality after re-exploration. In order to better screen critically ill patients after unplanned re-exploration thoracotomy, effectively improve the level of clinical treatment and improve the prognosis of patients.

## Materials and methods

### Clinical data

**Fig. 1:**
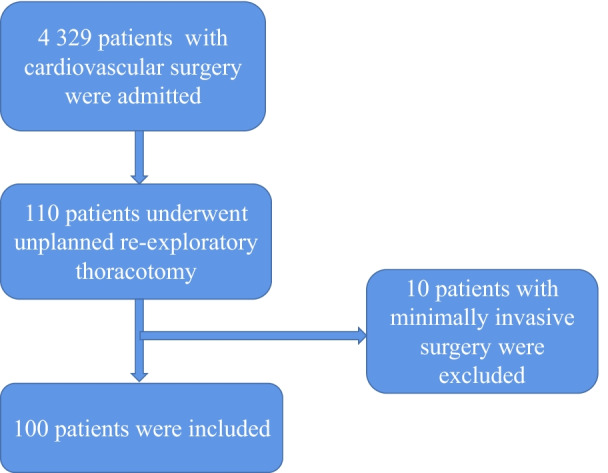
Schematic diagram of patients included in this study

The consecutive data of patients undergoing cardiac and aortic surgery in our hospital from May 2010 to May 2020 were retrospectively analyzed. Inclusion criteria: (1) underwent cardiac and aortic surgery; (2) remained in the ICU after surgery; (3) underwent unplanned re-exploration surgery. Exclusion criteria: (1) undergoing minimally invasive surgery, including robotic cardiac surgery; (2) planned re-exploration. Unplanned re-exploration refers to the need for emergency thoracotomy for re-exploratory thoracotomy after cardiovascular surgery due to bleeding, cardiac tamponade, ventricular fibrillation, etc. A planned re-exploration procedure is when cardiac surgeons delay closing the chest for various reasons, and then close the chest when the patient's condition is stable. During the period, a total of 4 329 patients with cardiac and aortic surgery were treated, including 110 patients admitted to the ICU after unplanned re-exploration after cardiovascular surgery, and 10 patients with minimally invasive surgery were excluded, as shown in Fig. 1. Therefore, a total of 100 patients were included in this study. There were 77 males and 23 females, with an age of (55.1 ± 15.2) years (range: 15 to 75 years) and a body mass index of (22.2 ± 3.0) kg/m^2^ (range: 17.1 to 35.2 kg/m^2^). Fifty-five patients had a history of hypertension, and 17 patients had a history of diabetes.

Informed consent and ethical approval were waived for this study, which did not contain identifiable data from patients.

### Research methods

#### Data collection

Demographic characteristics, surgical information, postoperative information, and perioperative complications of these patients were collected. Demographic characteristics included age, gender, body mass index, history of hypertension and diabetes. Surgery information includes operation time, operation method (including coronary artery bypass surgery, valve surgery, aortic surgery and other surgeries), whether it is secondary surgery, whether it is emergency surgery, and whether it is external bypass surgery. Postoperative information includes the reason and time of re-exploration and operation time of re-exploration, liver and kidney function indicators (such as serum creatinine, urea nitrogen, AST, ALT, total bilirubin and direct bilirubin) of the patient within 48 h, the worst blood lactate value and blood potassium value within 24 h, whether the intra-aortic balloon pump is needed (IABP), the need for extracorporeal membrane oxygenation (ECMO) or continuous renal replacement therapy (CRRT). Complications before and after re-exploration, mainly including cardiac insufficiency (New York Heart Association grade III–IV), acute kidney injury, perioperative liver insufficiency, respiratory insufficiency, pulmonary and bloodstream infections, craniocerebral complications (brain infarction, cerebral hemorrhage, coma, delirium and confusion), atrial fibrillation and ventricular fibrillation.

#### Establish an in-hospital mortality assessment model

The end point of observation was inpatient death. The differences between the variables in the survival group and the death group were compared, and the variables with significance in univariate analysis were included in the Logistic regression model for multivariate analysis to identify the prognostic factors affecting in-hospital death of patients, and a scoring model was constructed based on these prognostic factors.

#### Test the evaluation ability of the model

The receiver operating characteristic curve of the evaluation model was drawn, the area under the curve (AUC) was calculated, and the model performance was evaluated by the goodness-of-fit test.

### Statistical analysis

SPSS 22.0 statistical software was used to analyze the data. Quantitative data with normal distribution were expressed as mean ± standard deviation, and independent samples t-test was used for comparison between groups; quantitative data with non-normal distribution was expressed as M (QR), and comparison between groups was performed with nonparametric Mann–Whitney U test. Categorical data were expressed as frequencies and percentages, and comparisons between groups were performed using the χ^2^ test or Fisher's exact test. The variables with *P* < 0.05 in the univariate analysis were included in the logistic regression model for multivariate analysis to determine the prognostic factors of in-hospital mortality, and the receiver operating characteristic curve was drawn according to the results of the multivariate analysis and the AUC analysis was calculated. *P* < 0.05 indicated that the difference was statistically significant.

## Results

In our study, the in-hospital mortality rate for unplanned re-exploration was 2.5% (110/4 329) and the in-hospital mortality rate for enrolled patients was 26.0% (26/100). Univariate analysis showed that there were significant differences between the survival group and the non-survival group in terms of operation time, whether cardiopulmonary bypass surgery, and emergency surgery, etc., as shown in Table 1.

**Table 1:** Clinical data of patients underwent unplanned re-exploration after cardiovascular surgery

Group	Total case	Age (${\overline{\text{x}}}$ ± s, year)	Male	BMI (${\overline{\text{x}}}$ ± s, kg/m^2^)	Hypertesion	Diabetes	Emergency operation	Unplanned re-exploration	CPB	CABG	Valve surgery	Aortic surgery	Other operations	Operation time [M(Q_R_),h]
Survival	74	55.8 ± 15.4	57	22.1 ± 2.9	40	12	16	6	52	25	37	10	2	5.0 (2.5)
Non-survival	26	54.6 ± 15.1	20	22.6 ± 3.3	15	5	10	2	21	9	11	5	1	6.0 (4.0)
Statistics	–	t = 0.571	χ^2^ = 0.000	t = − 1.247	χ^2^ = 0.030	χ^2^ = 0.087	χ^2^ = 1.570	χ^2^ = 0.004	χ^2^ = 0.163	χ^2^ = 0.003	χ^2^ = 0.164	χ^2^ = 0.356	χ^2^ = 0.081	Z = − 4.406
*P* value	–	0.566	0.997	0.211	0.864	0.768	0.210	0.950	0.686	0.957	0.685	0.551	0.776	0.000

Values are presented as n or mean ± standard deviation;-suggests no data; other operations: including congenital cardiac surgery and cardiomyopathy surgery

*BMI* body mass index, *CPB* cardiopulmonary bypass, *CABG* coronary artery bypass grafting

Compared with patients in the survival group, patients in the non-survival group had a lower re-exploration rate due to higher drainage volume, but a higher re-exploration rate due to ventricular fibrillation and unexplained circulatory instability. In the non-survival group, the operation time of the unplanned re-exploratory thoracotomy was longer, and the use ratio of ECMO and CRRT was higher before and after the unplanned re-exploratory. There were differences between the two groups in the worst indicators of liver and kidney function before and 48 h after unplanned re-exploratory thoracotomy; moreover, the worst blood lactate values and worst blood potassium values were also different between the two groups before and 24 h after unplanned re-exploration thoracotomy, and the difference was statistically significant (Table 2). The incidence of cardiac insufficiency (New York Heart Association grade III–IV), respiratory insufficiency, acute kidney injury, perioperative liver insufficiency, blood infection, and craniocerebral complications in the non-survival group was significantly higher than that in survival group (Table 3).

**Table 2:** Comparison data of patients before and after the re-exploration

Variable	Group		Statistics	*P* value
	Survival	Non survival		
Reasons for re exploration				
Total case	74	26	–	–
Excessive drainage^a^	48 (64.9)	9(34.6)	χ^2^ = 2.719	0.099
Ventricular fibrillation	7 (9.5)	7 (26.9)	χ^2^ = 3.439	0.064
Pericardial tamponade	11 (14.9)	5 (19.2)	χ^2^ = 0.194	0.659
Unexplained circulatory instability	6 (8.1)	4 (15.4)	χ^2^ = 0.898	0.343
Other^b^	2 (2.7)	1 (3.8)	χ^2^ = 0.081	0.776
Re exploration time[M(Q_R_),h]	2.0 (1.0)	2.5(2.0)	χ^2^ = − 4.226	0.000
Laboratory indicators of pre exploration[M(QR)]				
Serum creatinine (mmol/L)	81.8 (36.7)	104.5 (79.2)	χ^2^ = − 4.596	0.000
Urea nitrogen (mmol/L)	7.0 (3.6)	8.3 (6.7)	χ^2^ = − 2.707	0.007
AST(U/L)	28.0 (30.0)	30.0 (37.5)	χ^2^ = − 2.532	0.011
ALT(U/L)	21.0 (23.5)	53.0 (164.0)	χ^2^ = − 5.036	0.000
Total bilirubin (umol/L)	18.8 (15.0)	22.2 (24.4)	χ^2^ = − 2.672	0.008
Direct bilirubin (umol/L)	4.5 (8.5)	7.3 (13.2)	χ^2^ = − 2.991	0.003
Lactic acid(mmol/L)	5.2 (5.6)	7.7 (9.8)	χ^2^ = − 3.623	0.000
Serum K^+^(mmol/L)	4.1 (0.7)	4.4 (0.8)	χ^2^ = − 3.351	0.000
Laboratory indicators of after exploration[M(Q_R_)]				
Serum creatinine (mmol/L)	102.6 (73.6)	197.7 (93.2)	χ^2^ = − 7.081	0.000
Urea nitrogen (mmol/L)	11.0 (7.8)	14.0 (6.9)	χ^2^ = − 3.701	0.002
AST(U/L)	72.0 (142.0)	681.0 (1591.2)	χ^2^ = − 7.382	0.000
ALT(U/L)	34.0 (75.3)	281.5 (900.5)	χ^2^ = − 8.184	0.000
Total bilirubin (umol/L)	36.0 (27.6)	50.9 (61.2)	χ^2^ = − 3.791	0.000
Direct bilirubin (umol/L)	15.2 (15.0)	22.2 (39.7)	χ^2^ = − 4.371	0.000
Lactic acid(mmol/L)	6.8 (6.1)	17.0 (18.2)	χ^2^ = − 7.545	0.000
Serum K^+^(mmol/L)	4.3 (0.7)	4.5 (1.1)	χ^2^ = − 2.243	0.025

^a^Shows that the chest drainage volume in the first hour after surgery is > 500 ml, or the chest drainage volume in the first 2 h is greater than 800 ml, or the chest drainage volume in the first 3 h is greater than 900 ml, or the chest drainage volume in the first 4 h is greater than 1000 ml, or suddenly Heavy bleeding or correction of blood coagulation function is still heavy bleeding; ^b^shows including low cardiac output syndrome, perioperative myocardial infarction.-shows no data.

**Table 3:** Comparison of the auxiliary treatmenst and complications of patients after re-exploration

Group	Total cases	IABP	ECMO	CRRT	Cardiac insufficiency	Respiratory insufficiency	AKI	PLI	Lung infection	Bloody infection	Craniocerebral complications
Survival	74	26	3	10	15	4	15	12	11	1	15
Non-survival	26	12	8	15	17	5	18	17	5	2	12
χ^2^ value	–	0.430	10.183	10.489	8.121	3.544	9.133	10.765	0.194	2.433	3.449
*P* value	–	0.512	0.001	0.001	0.004	0.060	0.003	0.001	0.659	0.119	0.063

Values are presented as numbers

*IABP* intra-aortic balloon pump, *ECMO* extracorporeal membrane oxygenation, *CRRT* continuous renal replacement therapy, *AKI* acute kidney injury, *PLI* perioperative liver insufficiency

– Shows no data

Statistically significant variables in the univariate analysis were included in the logistic regression model for multivariate analysis. The results showed that the worst blood creatinine value within 48 h before re-exploration, the worst blood lactate value within 24 h after re-exploration, cardiac insufficiency, respiratory insufficiency, and acute kidney injury were prognostic factors for in-hospital death in patients with unplanned re-exploration thoracotomy (Table 4). Based on these prognostic factors, an assessment model for in-hospital mortality in patients with unplanned re-exploration after cardiovascular surgery was constructed. The area under the receiver operating characteristic curve of the model was 0.910, indicating a good evaluation efficiency (χ^2^ = 4.153, *P* = 0.762).

**Table 4:** Prognostic factors of hospital mortality in patients with unplanned re-exploration after cardiovascular surgery

Variable	*B* value	*Wald* value	*df*	*OR* value	95% *CI*	*P* value
Re-exploratory time	0.286	6.758	1	1.392	1.108–1.748	0.009
Worst creatinine within 48 h before re-exploration	0.010	10.769	1	1.010	1.004–1.016	0.001
Worst creatinine within 24 h after re-exploration	0.121	23.582	1	1.124	1.070–1.181	0.000
Cardiac insufficiency	1.461	16.147	1	4.582	2.222–9.447	0.000
Acute kidney injury	1.142	8.504	1	4.171	1.894–9.187	0.004
Respiratory insufficiency	1.288	6.297	1	2.812	1.019–7.762	0.012

In addition, patients were divided into group A (patients with ventricular fibrillation + unexplained circulatory instability) and group B (patients with high drainage volume + cardiac tamponade + other causes) according to the reasons for unplanned re-exploration thoracotomy. The time of unplanned re-exploration thoracotomy in group A was 3.0 (3.0) h, which was longer than that in group B, which was 2.0 (0.5) h (*Z* = − 5.929, *P* = 0.000); the ICU stay time was 5 (7) d, which was longer than that in group B, which was 3 (5) d (*Z* =  − 2.148, *P* = 0.032); mechanical ventilation time was 111.0 (170.6) min, longer than 76.0 (101.3) min in group B (*Z* =  − 2.065, *P* = 0.039).

## Discussion

Cardiac and aortic surgery is the mainstay of treatment for cardiovascular disease, and postoperative bleeding and cardiac arrest are the main complications leading to early postoperative death or serious adverse events in patients. When these complications are difficult to correct with conservative treatment, patients often require unplanned re-exploration thoracotomy to avoid further deterioration. Unplanned re-exploration thoracotomy will bring a large number of allogeneic blood products to patients, increase the risk of mediastinal and deep soft tissue infection, etc. [8–10], which is a prognostic factor for patients with severe postoperative complications. Surgical trauma caused by unplanned re-exploration thoracotomy and volume control performed on patients during the perioperative period make the body prone to cardio-renal dysfunction, respiratory insufficiency, and even multiple organ dysfunction, which seriously affects the prognosis of patients.

In this study, the unplanned re-exploration operation time of the non-survival group was significantly higher than that of the survival group. Patients in the non-survival group had a longer operative time for re-exploratory thoracotomy, mainly because of ventricular fibrillation and unexplained circulatory instability. The results of some studies showed that the length of operation time was closely related to the risk of postoperative complications. The risk of complications increases with each additional hour of operative time [11–13]. However, in addition to the surgical method itself, the severity of the patient’s condition and the skill of the surgeon can also affect the duration of surgery [14]. It is necessary to further explore the reasons for the prolonged operation time in the future to better elucidate its relationship with patient prognosis.

Blood lactate level is also a prognostic factor for in-hospital mortality after cardiac surgery, and can more sensitively reflect the state of early hypoperfusion and hypoxia [15, 16]. The hemodynamics of cardiac surgery patients are not stable within 24 h after operation, which is prone to accumulation of lactic acid. Therefore, it is very important to closely monitor changes in lactate within 24 h after cardiac surgery. Once abnormal blood lactate is found, if the tissue ferfusion and oxygenation can be effectively improved in a short time, the clinical treatment effect of cardiac surgery patients will be significantly improved.

Acute kidney injury is a common serious complication after cardiac surgery and an independent prognostic factor of in-hospital mortality after cardiac surgery. Serum creatinine is closely related to changes in renal function after cardiac surgery. A slight increase in this index (> 0.017 mmol/L) will significantly prolong hospital stay and mortality [17, 18]. Our results showed that the worst serum creatinine value in the non-survival group was significantly higher than that in the survival group within 48 h before unplanned re-exploration, suggesting that monitoring serum creatinine in the perioperative period has a certain value. The area under the curve of the evaluation model constructed based on the above prognostic factors was 0.910, and the evaluation efficiency was good (2 = 4.153, *P* = 0.762), which further indicated that these prognostic factors had a higher evaluation value on in-hospital mortality in patients with unplanned re-exploration thoracotomy. In addition, there was a good fit between the data and patient outcomes. Identifying the prognostic factors of in-hospital mortality in patients can effectively guide us to timely screen critically ill patients after cardiovascular surgery, adjust clinical treatment strategies in a timely manner, and improve patient prognosis.

Of the 100 patients in this study, 25 were not scheduled for reexploration due to unexplained circulatory instability and ventricular fibrillation. Among them, 48.0% (12/25) patients died. The case fatality rate (48.0%) of this group (group A) was much higher than the case fatality rate [18.7% (14/75)] of patients with unplanned re-exploration due to large drainage and other reasons (group B). Compared with group A, the purpose of unplanned re-exploratory thoracotomy in group B was clear. Through timely and effective secondary thoracotomy to stop bleeding and placement of a drainage tube, the symptoms of the patients could be effectively relieved and the prognosis improved. When the patient undergoes a second exploratory thoracotomy due to circulatory instability or ventricular fibrillation caused by an unknown reason, the purpose and method of the operation before re-exploration are not clear. Secondary exploratory thoracotomy is often only used as a rescue operation. It is necessary to choose cardiopulmonary resuscitation, application of mechanical assistance, or another surgical treatment such as coronary artery bypass grafting according to the patient's intraoperative situation, which makes the re-operation time relatively prolonged and seriously affects the prognosis of patients. However, since this study only included patients who underwent secondary thoracotomy due to ventricular fibrillation or circulatory instability, the pros and cons of the treatment results compared with patients who did not undergo secondary thoracotomy are still uncertain, and further analysis is needed in the future. Clarifying the purpose of surgery and the reason for re-exploration has high clinical value for evaluating the patient's condition and formulating precise treatment plans.

This study still has some limitations. First of all, whether anticoagulant drugs are used before re-exploration, the type of anticoagulant drugs used and the time of application have a greater impact on postoperative bleeding and the prognosis of patients [19, 20]. In addition, due to the lack of original data, the corresponding analysis of anticoagulant drugs could not be performed in this study. Secondly, this study is a single-center retrospective study with a small sample size, which reduces the scientific nature of the research results. In the future, we need to collect more complete case data while expanding the sample size, and further verify the results through multi-center studies.

## Conclusions

The time of the second thoracotomy, the worst serum creatinine value within 48 h before the exploration, the worst lactate value within 24 h after the exploration, cardiac insufficiency, respiratory insufficiency, and acute kidney injury were the main prognostic factors for in-hospital death in patients with unplanned re-exploration. Identifying the prognostic factors of in-hospital death in patients with unplanned re-exploration can effectively guide clinicians to screen high-risk patients after cardiovascular surgery, adjust clinical treatment strategies in a timely manner, and improve patient outcomes.

## Data Availability

The datasets used and/or analysed during the current study are available from the corresponding author on reasonable request.

## Abbreviations

AUC: Area under the curve
ALT: Alanine aminotransferase
AST: Aspartate aminotransferase
ICU: Intensive Care Unit
IABP: Intra-aortic balloon pump
ECMO: Extracorporeal membrane oxygenation
CRRT: Continuous renal replacement therapy
